# Discovering privileged topologies of molecular knots with self-assembling models

**DOI:** 10.1038/s41467-018-05413-z

**Published:** 2018-08-03

**Authors:** Mattia Marenda, Enzo Orlandini, Cristian Micheletti

**Affiliations:** 1 SISSA, International School for Advanced Studies, via Bonomea 265, I-34136 Trieste, Italy; 2 Dipartimento di Fisica e Astronomia “Galileo Galilei”, sezione INFN, Università degli Studi di Padova, via Marzolo 8, I-35131 Padova, Italy

## Abstract

Despite the several available strategies to build complex supramolecular constructs, only a handful of different molecular knots have been synthesised so far. Here, in response to the quest for further designable topologies, we use Monte Carlo sampling and molecular dynamics simulations, informed by general principles of supramolecular assembly, as a discovery tool for thermodynamically and kinetically accessible knot types made of helical templates. By combining this approach with the exhaustive enumeration of molecular braiding patterns applicable to more general template geometries, we find that only few selected shapes have the closed, symmetric and quasi-planar character typical of synthetic knots. The corresponding collection of admissible topologies is extremely restricted. It covers all known molecular knots but it especially includes a limited set of novel complex ones that have not yet been obtained experimentally, such as 10_124_ and 15*n*_41185_, making them privileged targets for future self-assembling experiments.

## Abstract (web summary)

Only a few different types of supramolecular knots have been synthesized so far. Here the authors use Monte Carlo sampling, molecular dynamics and combinatorics to discover new knot types made of identical templates.

## Introduction

In the past two decades there have been major breakthroughs in understanding the principles of directed self-assembly^1–10^, as well as for the synthesis of molecules with complex topologies^11–14^. Besides being appealing from a fundamental research perspective, molecular knots and links have versatile applications^11,14–16^, from anion sensing and assisted catalysis^17–19^, to seeding weaving structures^20^ to their potential as molecular nano-cages^21^, functionalizable nanoscale scaffolds^22–24^, and the synthesis of polymers with special properties^25–31^. The challenges encountered in designing and then obtaining knotted molecular architectures lie in the numerous concurrent physico-chemical mechanisms that need to be balanced and steered. These include the length, thickness, intrinsic curvature and relative spatial orientation of the ligands and, above all, a judicious choice of the target topology^32–37^. Not all knot types are, in fact, equally designable neither a priori nor in practice^38^.

Until recently^39^, the repertoire of addressable molecular topologies consisted of only the three simplest knot types: the trefoil^40^, the figure-of-eight^13^ and the pentafoil knots^41^, as well as the first fundamental link types^15,35,42–44^. The 3_1_ and 5_1_ torus knots were assembled out of linear or circular double helicates whose crossings were templated by metal ions^40,41^. The 4_1_ twist knot was instead assembled from a solution of organic flexible-rigid building blocks^13^. Independently of the specific design strategy, the signature characteristics of these synthetic knots are two: cyclic symmetry and quasi-planar geometry.

In parallel with these experimental endeavours, theoretical and computational models have increasingly being used to optimise supramolecular design strategies and identify novel addressable topologies. To this end, our recent contribution was to devise general models based on self-assembling processes^5,45,46^ where a suitable shaping of the rigid building blocks boosted the spontaneous assembly of knotted or linked constructs^38,47^. This minimalistic approach allowed for identifying a complex eight-crossing knot, the 8_19_ torus knot, as one of the most designable undiscovered knot types and hence the ideal next target of designed synthetic topologies. In the recent experiment of ref. ^39^, Danon et al. have precisely succeeded in realising this very same type of 8_19_ molecular knot, thus underscoring the predictive power and reliability of general models of topological self-assembly.

At the same time,  no simple design strategy exists for identifying  additional privileged topologies obtainable with supramolecular techniques. 

Motivated by these observations, here we use models and extensive stochastic simulations, informed with general principles of supramolecular assembly, for a systematic survey of thermodynamically and kinetically accessible knot types and use it to discover novel topologies that have not yet been obtained experimentally.

Our main finding is that the unsupervised selection procedure, besides recovering  previously synthesised molecular knot types (3_1_, 4_1_, 5_1_ and 8_19_), can indeed identify novel privileged topologies such as the 10_124_ and 15*n*_41185_ prime knots. These topologies have a higher complexity than those realised before and yet they share the same underlying architectural simplicity: they are quasi-planar, circular-symmetric and can be formed by only five templates of helical shape. To extend considerations beyond strictly helical templates, we survey the combinatorics of molecular braiding diagrams and show that the two mentioned topologies are part of a wider population of cyclic-symmetric knots that we propose as privileged addressable topologies for future molecular constructs. These include the 8_18_ topology that was reported experimentally^48^ after submission of this study.

## Results

### Model system

The templates, or building blocks, used for our self-assembling survey are modelled after helicates, which are commonly used in supramolecular synthetic chemistry^17,40,43,49^.

Specifically, we consider helical fragments of unit radius, angular span *α* and pitch *h*. They are discretised as chains of beads with attractive patches at their termini, see Methods. The steric repulsion of the beads and the attractive patchy interactions allows the templates to attach one to another in a stringlike fashion^50^.

The helical shape of the templates, with its non-planar curved geometry, is ideally suited for our survey because it combines the simplicity of its mathematical parametrisation with the possibility to establish complex three-dimensional structures either open or closed. For instance, prior to its elegant experimental realisation^39^, the 8_19_ knot had been found to be designable precisely using helical templates in simulations of aspecific assembly processes^38^.

Here, we instead consider assemblies formed under specific constraints, common to known molecular knot types, by helical templates with angular span and pitch varying respectively in the ranges 1.4*π* ≤ *α* ≤ 1.9*π* and 0.1 ≤ *h* ≤ 2.0, the unit of length being the helical radius. Combinations of templates with different chiralities are considered, too.

### Monte Carlo discovery of novel addressable topologies

**Fig. 1:**
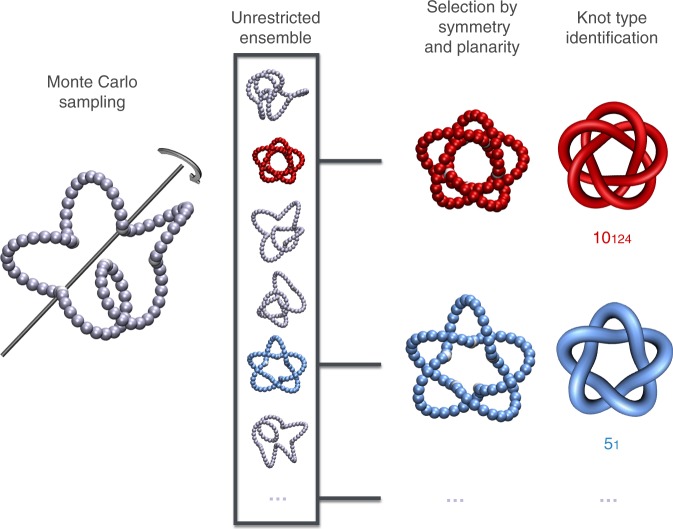
Discovery of privileged topologies. The two-tier strategy to detect admissible topologies consists of an initial Monte Carlo sampling of closed constructs made of *n*_*T*_ = 3, 4, 5 helical templates followed by an a posteriori selection of the quasi-planar, cyclic-symmetric and knotted instances. In this *n*_*T*_ = 5 example, the ensemble generated with unrestricted crankshaft moves includes two such instances: the 10_124_ and the 5_1_ knots. For visual clarity, here and in other figures the attractive patches joining the helical templates (discretised as chains of beads) are shown as white beads larger than their actual size

We proceeded in two steps, as sketched in Fig. 1. First, we explored the repertoire of thermodynamically accessible topologies of closed constructs formed by a fixed number of helical templates, *n*_*T*_. Next, to identify the candidate shapes of feasible realisation, we select a posteriori those having the signature features of existing synthetic molecular knots: cyclic symmetry and quasi-planarity.

For the first step, we used a Monte Carlo procedure to explore the equilibrium conformational space accessible to constructs made of *n*_*T*_ = 3, 4 and 5, templates, as typical for known molecular knots. For a given value of *n*_*T*_, we independently generated 1000 constructs for each template shape, i.e., (*h*, *α*), and possible combinations of left- of right-handed template chiralities. The second step involved identifying the conformers with the sought cyclic symmetry. This was established by requiring that the constructs admitted one or more precise structural superpositions^51^ with their circular permutants, see Methods.

**Fig. 2:**
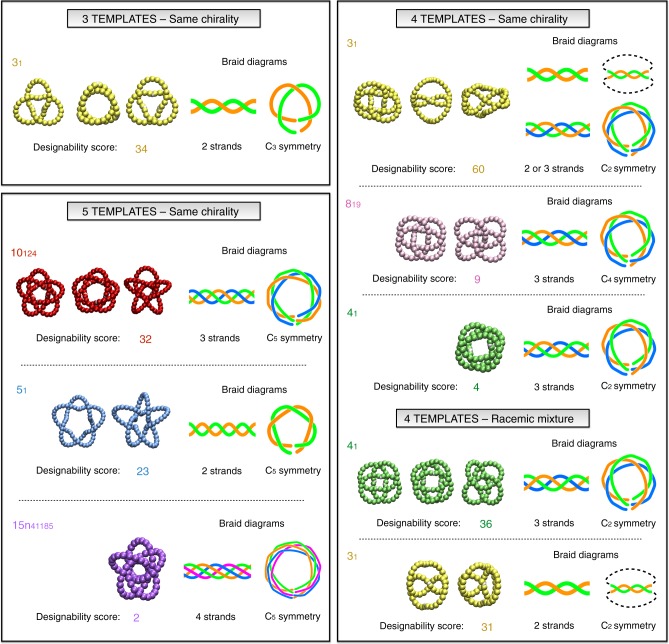
Exhaustive repertoire of discovered privileged topologies. The graphical table provides the complete repertoire of non-trivial topologies that can be realised with up to five identical helical templates (of same or opposite chirality) in the form of cyclic-simmetric and quasi-planar constructs. For each knot type we show one or more representative conformers (grouped by the number of templates) and the corresponding linear and braid representations. The designability score is the number of points in the discretised (*h*, *α*) parameter space of helical template shapes for which that knot type is observed

Figure 2 summarises the outcome of this phenomenologically informed discovery procedure. It presents the repertoire of the different types of closed, cyclic-symmetric knotted constructs found across the entire range of shapes and chiralities of the building blocks.

The graphical table of Fig. 2 displays the structural representatives of each knot type. Strikingly these are all inherently oblate, meaning that quasi-planarity is co-opted by the cyclic symmetry constraint and does not need to be enforced additionally. Their Cartesian coordinates can be found in Supplementary Data 1.

Each representative in Fig. 2 is accompanied by a number of key properties, namely the braid representation of the knot, in both linear and circular form, and the topological designability score. We define the latter as the number of distinct template shapes, i.e., distinct points in the discretised  (*h*,*α*) space that can assemble in that particular cyclic knot. This entropic measure is reflective of the designability of the knot that is the robustness to variations in template shapes. This ought to be a valuable quantitative element for guiding the design of novel molecular topologies. The regions in the (*h*, *α*) parameter space where the various knot types occurr, are highlighted in the topological state diagram of Supplementary Figs. 1 and 2.

The main result of Fig. 2 is that, across the wide range of template shapes and combinatorics of sampled constructs, the phenomenological selection is survived by only few privileged topologies.

These cover all knot types that have been synthesised so far and, above all, include different ones too. Entries in Fig. 2 that correspond to known molecular topologies are the 3_1_, 4_1_, 5_1_ and 8_19_ knots. The latter two are obtained with 5 and 4 templates, respectively, as in experimental realisations ^39,41^. Interestingly, the 3_1_ knot is observed in different geometries, including the ideal-like and the twisted one obtained with 3 and 4 templates, respectively, again as in the synthetic constructs^40,52^. These instances are all torus knots^53^.

The only non-torus knot in Fig. 2 is the 4_1_, or figure-of-eight knot. Interestingly, one notes that this twist achiral knot can be established with four templates, either with the same or opposite chirality. The latter, racemic combination is more suitable because it covers a significantly wider region of parameter space, see designability score and Supplementary Figs. 1 and 2, and yields more planar constructs, see Supplementary  Data 1 with coordinates. The 4_1_ topology has been experimentally obtained before, too, and with the same number of templates, though the building blocks were not helicates but flexible diblock modules^13^. The 4_1_ instance in Fig. 2 therefore makes the important point that this topology would be realisable with non-negligible probability also by using metal templating technique and helicates, but the latter should ideally be of opposite chirality. This is a feature that, to our knowledge, has not yet been systematically explored.

The fact that these previously obtained molecular knots are all included in the graphical table, gives confidence in the feasibility to synthesise in the future the two novel topologies appearing in Fig. 2.

These are the 10_124_ and 15*n*_41185_ knots. Though being made by only *n*_*T*_ = 5 templates (same chirality), like the 5_1_ synthetic knot, these topologies are more complex than all known synthetic knots obtained so far. In fact, it is arresting to observe that, out of the millions of prime and composite knots with of up to 15 crossings, those feasible with a handful of cyclically arranged helical templates are only 6.

The 10_124_ topology emerges as a particularly designable cyclic knot, because it can be obtained with tens of different template shapes (Supplementary Fig. 2). It is intriguing that this topology is among those that are only rarely populated in aspecific self-assemblies^38^, i.e., without cyclic symmetry or quasi-planarity, and appears, with other instances, in energy-minimizing arrangements of dipolar particles^54^.

The 15-crossing knot is an even more remarkable example of sophisticated interplay of geometry and topology, with as many as 15 crossings established by 5 templates only. It forms abundantly for a specific template shape, the one involved in the construct shown in Fig. 2, and more limitedly for a second template geometry, see Supplementary Fig. 2.

Of these two novel complex topologies, we therefore single out 10_124_ as the most promising one to be realised with current self-assembly strategies.

### Kinetic accessibility of self-assembling knotted constructs

**Fig. 3:**
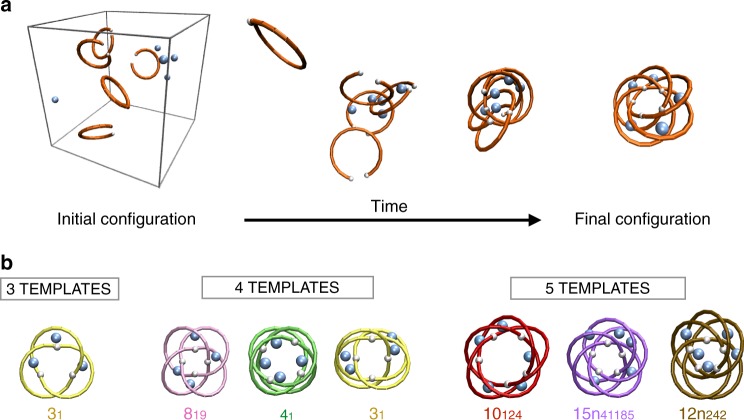
Kinetically accessible topologies from self-assembly simulations. **a** Series of snapshots from a molecular dynamics simulation where five helical templates and an identical number of coordinating particles self-assemble spontaneously into a cyclic-symmetric 15*n*_41185_ knot. This topology and the 10_124_ one emerge as two privileged knot types that have not yet been obtained experimentally. **b** Repertoire of cyclic-symmetric quasi-planar knotted constructs that recurrently form in self-assembly simulations. The 12*n*_242_-knotted construct is nearly cyclic-symmetric and is included for its high statistical incidence. For visual clarity the templates are represented with their centerline, omitting the constitutive beads

To test the kinetic accessibility of the topologies discovered by the Monte Carlo sampling, we performed hundreds of self-assembly simulations varying templates geometries. We used Langevin molecular dynamics (MD) to evolve dispersions of patchy helical templates in various conditions, see Fig. 3a, Supplementary Fig. 3 and Methods. To mimic helicate coordination by templating metal ions^55^, we also considered adding several coordinating particles (same diameter of the beads) that do not bind to each other but can attract, and hence bring together, two or more templates.

By exploring combinations of varying number of templates and coordinating particles, from few to hundreds, see Supplementary Fig. 3, we established that the simplest self-assembling strategy for producing cyclic-symmetric quasi-planar constructs was to focus on systems with an equal number of templates and coordinating particles, *n*_*T*_ = 3, 4, 5 at a suitable density, see Methods.

With these systematic self-assembly simulations, we established that all privileged topologies listed in Fig. 2 except for the 5_1_ knot (because of its large contact angle, see Supplementary Fig. 4), are indeed kinetically accessible. Their representative structures formed by self-assembly are shown in Fig. 3b, while their cartesian coordinates are available in Supplementary  Data 1. These conformers include the novel complex target topologies, 10_124_ and 15*n*_41185_. This reinforces their general viability as new targets for molecular constructs with addressable topology.

Interestingly, besides the topologies of Fig. 2, we recurrently observed the formation of the 12*n*_242_ topology, as shown in Fig. 3b and Supplementary Table 1. This knotted structure is self-assembled from five templates and differs from the 15*n*_41185_ by a localised defect in the otherwise regular pattern of over- and under-crossings. From its abundance, we conclude that the 12*n*_242_ topology, though not cyclic-symmetric, might be obtainable as a likely by-product of the target 15*n*_41185_ knot.

### Enumerative survey of cyclic-symmetric entangled constructs

The geometrical and topological repertoire spanned by the constructs of Figs. 2 and 3b can be recapitulated in terms of only two key parameters: the number of templates, *n*_*T*_, which is equal to the number of crests on each edge of the corresponding braid, and the number of strands in the braid itself, *n*_*S*_. The latter matches the number of complete turns around the cyclic symmetry axis, but is more general because it can be defined for non-cyclic knots too. Both parameters have, in fact, proved useful in systematic explorations of possible synthetic topologies^39^, arguably because they reflect different aspects of the practical difficulty of their realisation.

Classifying conformers in terms of *n*_*T*_ and *n*_*S*_ is an apt way to code their structure independently of the specific geometry of their templates. For this reason, a systematic search of the (*n*_*T*_, *n*_*S*_) parameter space can reveal additional addressable topologies besides those in Figs. 2 and 3b made of strictly helical building blocks or, else, rule out the existence of alternative ones.

Accordingly, we completed the survey with an exhaustive enumeration of braids for different combinations of (*n*_*T*_, *n*_*S*_). For practical reasons, we limited the combinatorial search up to *n*_*T*_ = 7 (see Supplementary Fig. 11 for *n*_*T*_ = 8, 9 and 10 too) and we assumed *n*_*T*_ ≥ *n*_*S*_ + 1, meaning that viable templates should cover less than a full turn when projected.

**Fig. 4:**
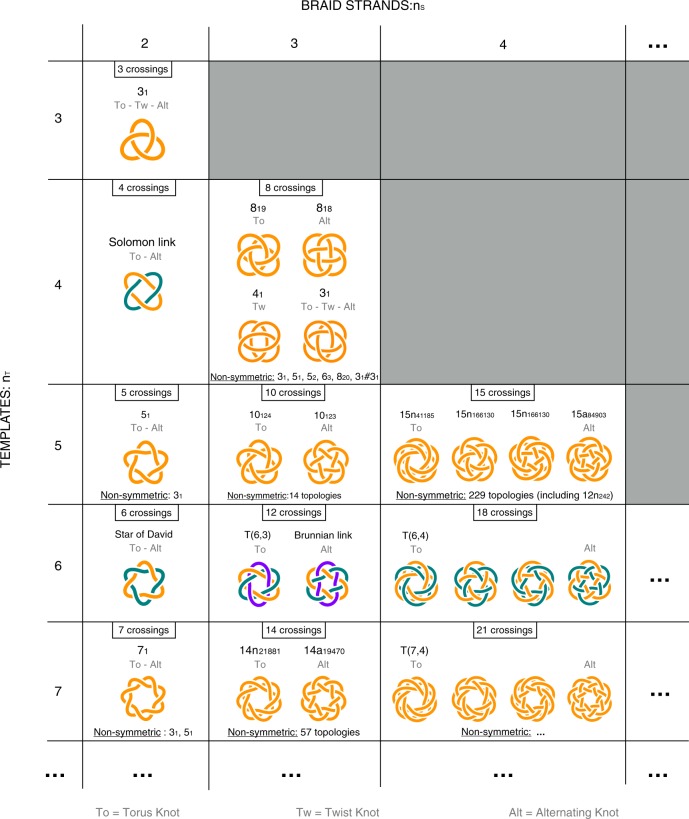
Graphical table of non-trivial topologies with planar cyclic-symmetric representations. Non-trivial knots and links with cyclic-symmetric planar representations are organised in terms of *n*_*T*_ and *n*_*S*_. The former is the number of constitutive templates, i.e. modular units with projections covering less than a full turn, which is taken equal to the number of crests on each edge of the corresponding linear braid; the latter is the number of strands in the braid itself. The number of projected crossings (an upper bound to the crossing number) is *n*_*T*_ × (*n*_*S*_ − 1) and is identical for various topologies obtainable with the same combination (*n*_*T*_, *n*_*S*_). For knots all inequivalent symmetric diagrams are shown, while for links only those with the highest degree of cyclic symmetry are presented for *n*_*T*_ = 6. Various non-symmetric instances are listed too. The complete list of knots is given in Supplementary Figs. 8–11

The results are shown in Fig. 4. Note that, since the search scheme based on braiding combinatorics is more general than the Monte Carlo exploration, the topologies listed for each (*n*_*T*_, *n*_*S*_) pair are not limited to those in Fig. 2. They include, for example, the 8_18_ and 10_123_ knots that, owing to their alternating character, are not realisable with a small number of templates if these have a strictly helical geometry. Moreover, when *n*_*T*_ and *n*_*S*_ are not mutually prime they include multicomponent constructs, such as catenanes and links. In such cases, the number of components corresponds to the greatest common divisor of *n*_*T*_ and *n*_*S*_.

For both knots and links in Fig. 4, the number of projected crossings is equal to *n*_*T*_ × (*n*_*S*_ − 1). Note that this geometrical measure of complexity, which does not necessarily coincide with the so-called minimal crossing number, is identical for all topologies realisable with the same number of templates and strands, (*n*_*T*_, *n*_*S*_). For instance, various 8-crossing knots are obtained for (*n*_*T*_ = 4, *n*_*S*_ = 3). The cyclic ones, shown in Fig. 4, are the 8_19_ and 8_18_ knots, with *C*_4_ symmetry, and the 3_1_ and 4_1_ ones, with *C*_2_ symmetry. Non-cyclic-symmetric instances are listed but not shown in Fig. 4 and include non-minimal representations of 5_1_ and 5_2_ topologies. Similarly, for (*n*_*T*_ = 5, *n*_*S*_ = 4) one obtains various 15-crossing knots: four cyclic ones, including the privileged 15*n*_41185_ topology, and many more acyclic ones, such as the 12*n*_242_ knot that recurred in self-assembling simulations, see Fig. 3b. Non-symmetric knots are listed in Supplementary Figs. 8–11.

The topology indexing scheme of Fig. 4 has a twofold implication.

First, it systematically recapitulates the repertoire of designable topologies in terms of two parameters with direct bearings on the complexity of their practical realisation: the number of templates and the number of strands. The key emerging point is that ranking topologies in terms of realisation complexity, by the number of templates and braided strand, subverts the canonical order of nominal topological complexity^56^. For instance, cyclic-symmetric realisations of knots with 7 minimal crossings, such as 7_1_, requires more templates than prime knots with 8 or 10 minimal crossings (e.g., 8_19_ or 10_124_). At the same time, complex knots with 8, 10, 14 and 15 crossings can be realised with fewer templates than topologies with 6 or 7 projected crossings. This stresses the fact that the expected difficulty of realisation does not necessarily parallel the nominal complexity, and hence reinforces a posteriori the necessity of systematic survey for judicious choices of target topologies^57^.

Second, it provides a systematic route towards discovering new designable topologies by extending the range of *n*_*S*_ and *n*_*T*_ even beyond the cases considered here. This search ought not to be aimed only at the simplest types of novel knots, because complex three-dimensional geometries would be even better suited for specific goals, such as realising molecular cages^21,58^. Arguably, S-shaped^34^ or otherwise wavy building blocks ought to be more suited than constant-curvature ones (including straight and helical templates) to produce the more intricate entries in Fig. 4, and particularly those with the largest number of braid strands, *n*_*S*_, for a given number of templates, *n*_*T*_.

## Discussion

In summary, we reported on a systematic discovery scheme for novel molecular topologies. The method combines Monte Carlo, molecular dynamics and braiding patterns enumeration and was used to single-out knot types that have the same signature features, notably cyclic symmetry and quasi-planarity, shared by all known synthetic molecular knots.

It was thus established that such repertoire of admissible topologies includes only a tiny fraction of all possible knot types. In particular, there are only 6 distinct cyclic-symmetric knot types that can be assembled with 5 or fewer helicate-like templates. Four of them, namely 3_1_, 4_1_, 5_1_ and 8_19_, have been previously obtained in a remarkable progression of synthesis strategies spanning two decades; the other two, instead, are yet to be realised experimentally. These correspond to the 10_124_ and 15*n*_41185_ knot types. With their ten and 15 minimal crossings, respectively, these topologies largely surpass previous ones in terms of complexity. At the same time, their characteristics of cyclic symmetry, quasi-planarity and kinetic accessibility makes them ideal targets of future molecular designs efforts.

Finally, by exhaustive enumeration of braid patterns, we found that only few and specific types of additional knots might be addressable by using either a larger number of helical templates, or few templates but with wavy or S-shaped geometries^34^, either rigid^37^ or with flexible termini^13^.

The simplest of these privileged topologies appear in Fig. 4. The shown targets include the 8_18_ topology, which was reported experimentally^48^ after submission of this study. This molecular knot was obtained with a non-minimal geometry, corresponding to (*n*_*t*_ = 8, *n*_*s*_ = 3) in the scheme of Fig. 4, see Supplementary Fig. 11. This match confirms the predictive capabilities of the enumerative scheme of Fig. 4.

The in silico exploration and optimization of templates shapes ought to be valuable also in supramolecular DNA assembling strategies^6,59–61^ that are characterised by a good control of the local curvature of templates as well as of their spatial coordination and binding pattern.

## Methods

### Building blocks

As building blocks for self-assembling structures we consider rigid helical fragments. The helices project a circle of unit radius and their pitch *h* is varied in the [0.1;2.0] range at 0.1 increments. The angular span of the fragments, *α* is varied in the [1.4;1.9]*π* range in steps of 0.1*π*. Both possible chiralities *χ* are considered: right (+1) or left (−1). The helical trace of the fragments is described by the parametric equation {*x* = cos(*αt*); *y* = sin(*αt*); *z* = *χht*;} where *t* ∈ [0, 1]. On this helical trace we then placed the centres of spherical beads with diameter equal to *σ* = 1/3. The centres are equispaced at a distance in the [*σ*, 2^1/6^*σ*] range, as needed to fit an integer number of beads along the contour. Following ref. ^38^ each template is functionalised with two small attractive patches (white spheres in Figures, enlarged for visual clarity) lying on the exposed surface of the terminal beads at the intersection point with the helical centerline.

### Monte Carlo sampling

A Monte Carlo procedure based on crankshaft moves is used to explore the conformational space of closed constructs made of *n*_*T*_ = 3, 4, 5 templates. These constructs are formed by identical rigid templates, i.e., same (*h*, *α*) geometry, joined at their patchy ends in a circular fashion, see Fig. 1. For each template geometry and template number, all combinations of left and right-handed chiralities are considered. In the Monte Carlo scheme, bonded templates are joined by exactly superposing the patches at their termini. The connectivity of the constructs is preserved by using unrestricted crankshaft moves that are hinged at randomly chosen pairs of these superposed patches, see Fig. 1. Being their spatial superposition kept fixed at all times, the patches, and the templates too, cannot detach and therefore no patch-patch attractive potential is introduced. All configurations are accepted, except those with steric clashes (overlapping beads of different templates) or with large (>*π*/4) contacting angles between consecutive templates, which are rejected. For each combination of template shape, (*h*, *α*), and chirality, we sampled 1000 configurations spaced by 5 × 10^4^ Monte Carlo (crankshaft) moves, a timespan larger than the typical autocorrelation time for the considered systems.

From the set of sampled conformations we then selected a posteriori those, if any, with approximate cyclic symmetry. To this end, we structurally aligned the constructs with their circularly permuted variants, and kept track of the corresponding root mean square deviation (RMSD) of the alignments^51^. The sought cyclic-symmetric constructs were identified from the atypically low RMSD, see Supplementary Fig. 5.

### Molecular dynamics simulations

We used Langevin molecular dynamics simulations to study the spontaneous self-assembly of helical templates into complex constructs. The system used in the MC approach is here enriched by a number of particles in solution that do not bind to each other but can attract, and hence coordinate, the templates. The role of these coordinating particles is to mimic the effects of metal ion templating^55^. Various simulations were carried out with a wide range of number (and relative size too) of templates and coordinating particles, from few to several hundreds, see Supplementary Fig. 3, as well as with different interaction parameters. These simulations were aimed at identifying the most suitable conditions yielding cyclic-symmetric quasi-planar constructs made of few templates. The choice of the specific parameters values was mostly guided by the need to make the assembly process computationally amenable.

The simplest conditions, those used for the production runs, are detailed below and correspond to systems with a small and equal number of templates and coordinating particles, *n*_*T*_ = 3, 4, 5, placed in periodic simulation boxes of side a few times larger than the templates’ projected diameters.

With exception of the small patches all other ones (templates beads and the coordinating particles) interact sterically through a truncated and shifted Lennard-Jones potential:

1 $$U^{{\mathrm{LJ}}} = 4{\kern 1pt} C_{\mathrm{LJ}}{\kern 1pt} \epsilon \left[ {\left( {\frac{\sigma }{d}} \right)^{12} - \left( {\frac{\sigma }{d}} \right)^6 + \frac{1}{4}} \right]\theta \left( {2^{1/6}\sigma - d} \right),$$

where $\epsilon$, the unit energy, is equal to the thermal energy of the system *K*_B_*T*, *σ* is the particle diameter, *d* is the distance of the particles’ centres, *C*_LJ_ = 100, and *θ* is the Heaviside function.

The patches, instead, interact via an attractive Gaussian potential well,

2 $$U^{{\mathrm{patchy}}} = - C_p{\kern 1pt} \epsilon {\kern 1pt} {\mathrm{exp}}\left( { - \frac{{d^2}}{{2\sigma _p^2}}} \right),$$

where *C*_*p*_ = 25 and its range *σ*_*p*_ = 0.1*σ*, as in ref. ^38^. The combination of these interaction terms results in an attractive directional potential well of depth ~18 *K*_B_*T* between helical templates, see Supplementary Fig. 6.

The attraction of the coordinating particles and the templates’ beads is described with a Yukawa-like potential

3 $$U^{{\mathrm{Yukawa}}} = - C_Y\frac{\sigma }{d}\epsilon {\kern 1pt} {\mathrm{exp}}\left( { - \frac{d}{{l_Y}}} \right),$$

where *C*_*Y*_ = 8 and *l*_*Y*_ = *σ*. A repulsive version of this potential (same form but opposite sign) is introduced between the coordinating particles to keep them apart.

Simulations were carried out in a periodic cubic box of size *L*, with the LAMMPS software package^62^ with default values for the beads mass and viscous friction and integration time step equal to 0.006*τ*_LJ_, where *τ*_LJ_ is the characteristic Lennard-Jones time.

For each value of *n*_*T*_ and template geometry we carried out 20 independent simulations at fixed template number density *ρ*_*T*_ ≡ *n*_*T*_/*L*^3^ = 0.0125, where *L* is in units of the helical radius. Note that the duration of each simulation was 264,000*τ*_LJ_, i.e., sufficiently long to observe spontaneous binding and assembling of templates, but still shorter than their typical unbinding time (~4,000,000 *τ*_LJ_, Supplementary Fig. 7).

### Knot analysis

For most constructs, the patterns of crossings in a two-dimensional projection was simple enough that the Alexander polynomial sufficed to identify the knot type^63^. For more complicated patterns, the Dowker code was compared against a look-up table for prime knots of up to 16 crossings using the Knotscape software package of J. Hoste and M. Thistletwaite available from www.math.utk.edu/morwen/knotscape.html.

### Data availability

The coordinates of the representative conformers shown in Figs. 2 and 3 are available as Supplementary Data 1.

The custom code for the Monte Carlo exploration is available from the authors. The code is made available freely for academic use and without user support.

## Electronic supplementary material

Supplementary Information

Description of Additional Supplementary Files

Supplementary Data 1
