# Supplementary material for: Discovering privileged topologies of molecular knots with self-assembling models
Source: Nat Commun. 2018 Aug 3;9:3051. doi: 10.1038/s41467-018-05413-z (PMC6076300; doi:10.1038/s41467-018-05413-z)
Supplement: Supplementary file 1 — Supplementary Information [file 41467_2018_5413_MOESM1_ESM.pdf]

Supplementary Information
Discovering privileged topologies of molecular knots with self-assembling models

M. Marenda *et al.*

Supplementary Note 1. Topological state diagrams

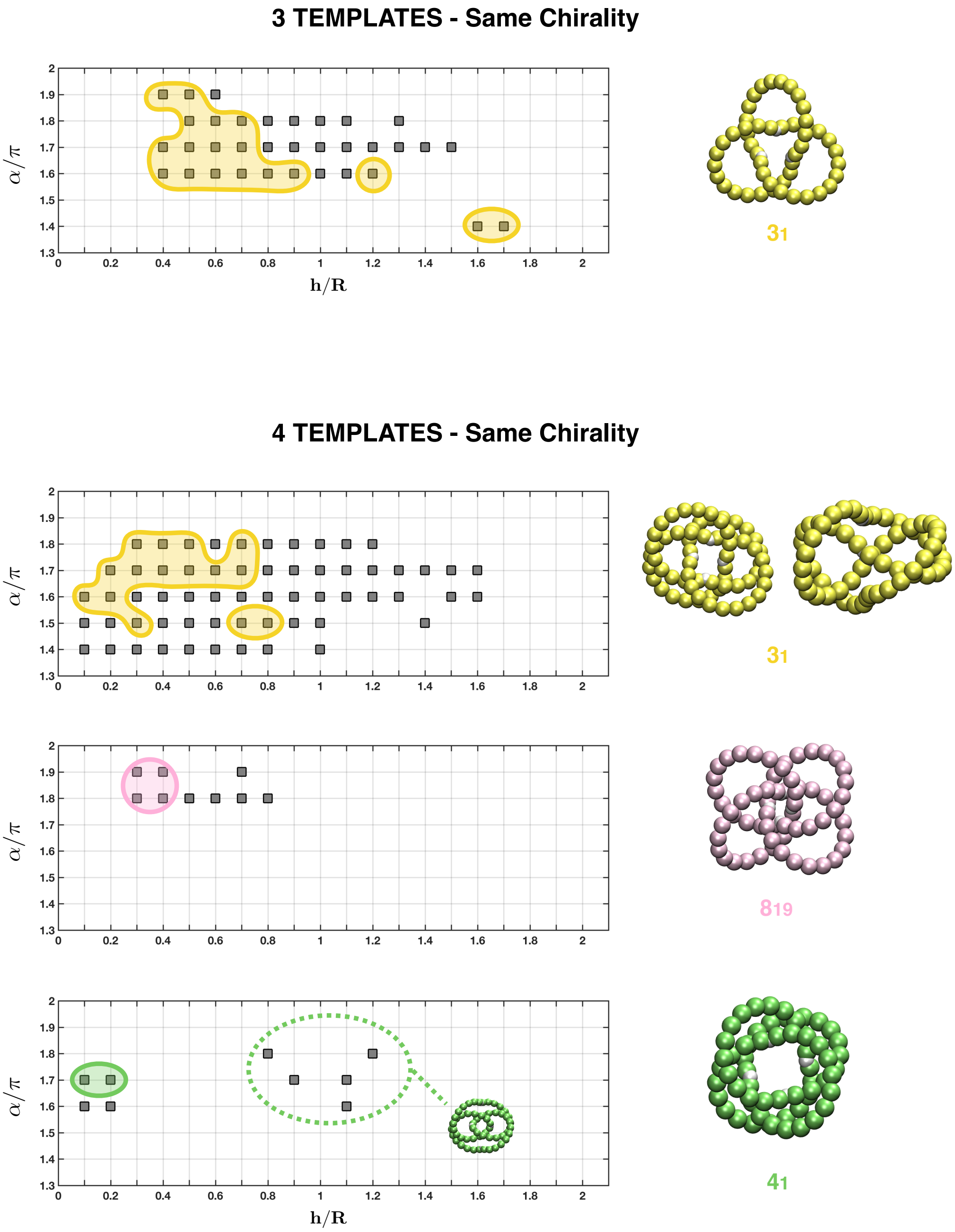

Supplementary Figure 1: Topological state diagram. The highlighted points in the discretized (h, α) parameter space mark the template shapes where one observes constructs $n_T = 3$ (top) and $n_T = 4$ (bottom) templates with the topologies sketched on the right. The highlighted regions include points where the indicated knots account for more than 1% of the MC-sampled constructs. The cases shown here are made of templates with the same chirality. The 4_1-knotted instances in the bottom panel mostly correspond to the geometry shown on the right, but there are also instances of an alternative geometry, shown inside the topological state diagram.

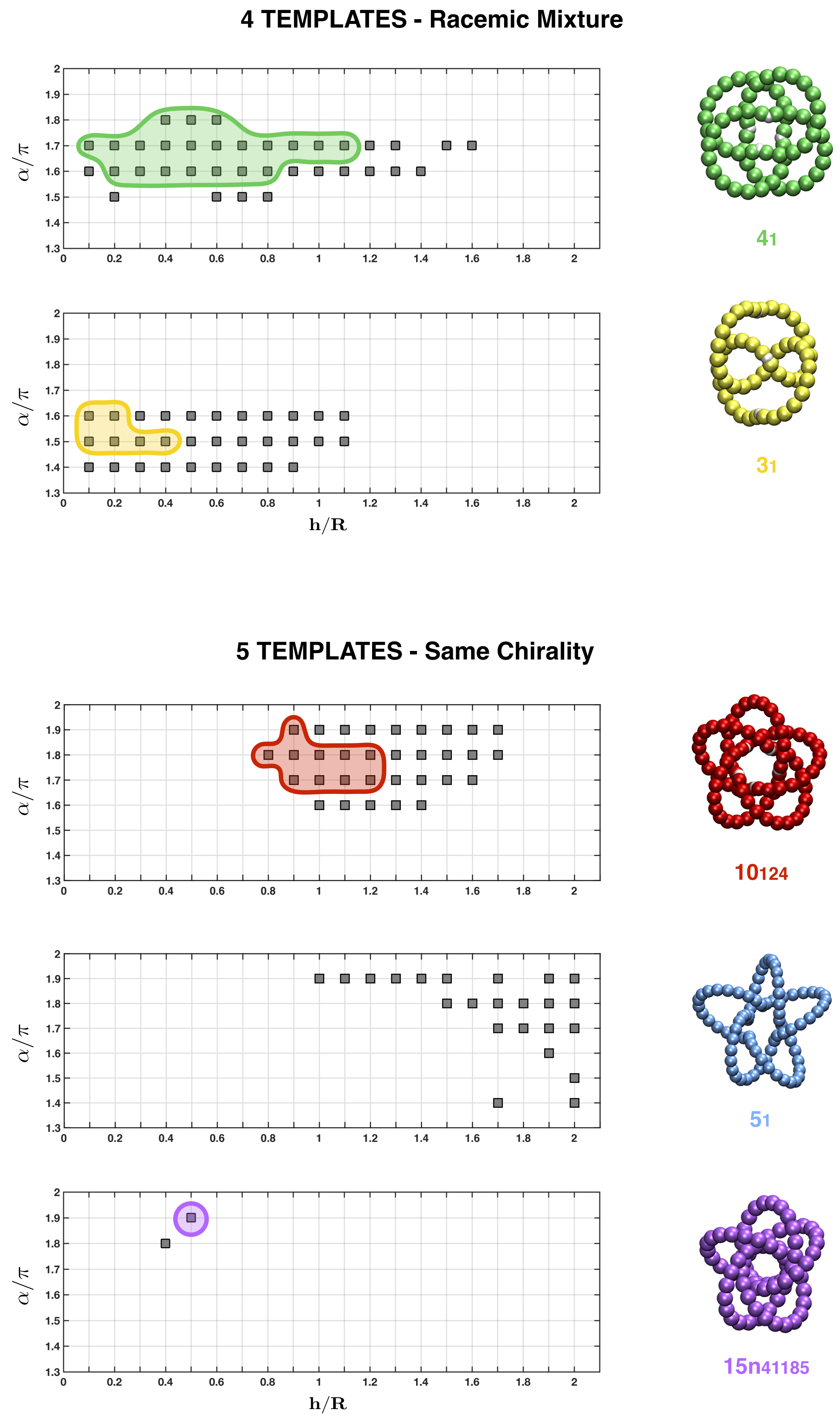

Supplementary Figure 2: Topological state diagram. The highlighted points in the discretized (h, α) parameter space mark the template shapes where one observes constructs $n_T = 4$ (top, racemic template mixture) and $n_T = 5$ (bottom, templates of same chirality) templates with the topologies sketched on the right. The highlighted regions includes points where the indicated knots account for more than 1% of the MC-sampled constructs.

Supplementary Note 2. Designability score of constructs obtained with self-assembly simulations

	3 templates	4 templates			5 templates		
**Topology**	3_1	8_{19}	4_1^*	3_1	10_{124}	$15n_{41185}$	$12n_{242}$
**Designability Score**	42	24	20	55	11	5	9

Supplementary Table I: Designability score for the symmetric (and quasi-symmetric) knots obtained in self-assembly of 3, 4 and 5 templates with shape parameters ($h \in [0.1, 2.0]$ and $\alpha \in [1.4, 1.9]\pi$).
(*) The 4_1 is assembled from a racemic combination of 2 left- and 2 right-handed templates.

Supplementary Note 3. Self-assembly in mixtures of hundreds of templates

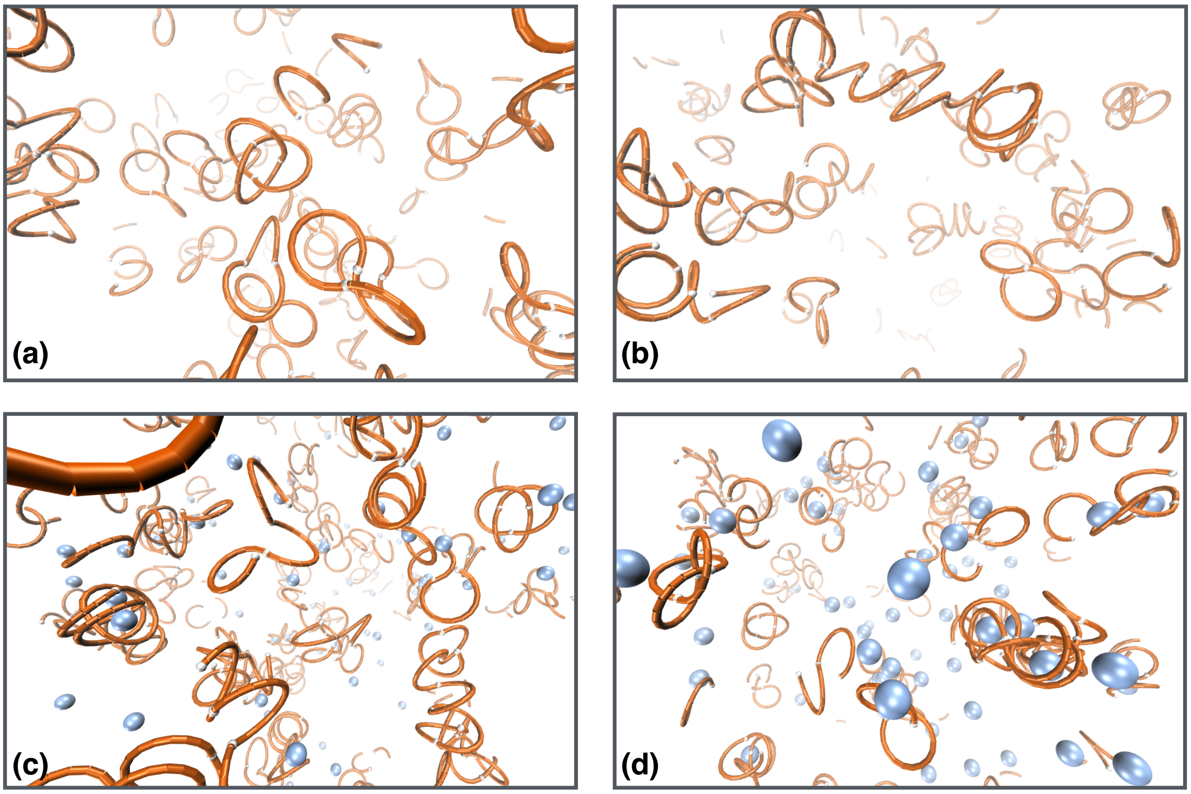

Supplementary Figure 3: Snapshots of molecular dynamics simulations for the self-assembly of 250 templates in various conditions. Self-assembly of templates without coordinating particles: **(a)** same chirality helical fragments with geometry $h = 1.0$ and $\alpha = 1.8\pi$ and helical fragments density 0.0125; **(b)** racemic mixture of helical fragments (ratio 50%) with geometry $h = 0.8$ and $\alpha = 1.7\pi$ and helical fragments density 0.0075. Self-assembly of templates with coordinating particles: **(c)** racemic mixture of helical fragments (ratio 50%) with geometry $h = 0.8$ and $\alpha = 1.7\pi$, helical fragments density 0.0125 and Yukawa parameters $C_Y = 10$ and $l_Y = 0.91\sigma$. The diameter of the coordinating particles in this example is 2σ, that is twice the size of the templates' beads. **(d)** Racemic mixture of helical fragments (ratio 50%) with geometry $h = 0.8$ and $\alpha = 1.7\pi$ and helical fragments density 0.0125; coordinating particles diameter 3σ, Yukawa parameters $C_Y = 5$ and $l_Y = 1.59\sigma$. For cases (c) and (d), the reference "contact distance" was suitably changed from the $2^{1/6}\sigma$ value in eq. (1) of the main text, which is appropriate only for the default case of equally-sized template beads and coordinating particles. Other parameters are set to their default values given in the Methods section of the main text.

Supplementary Note 4. Contact angles in 5_1-knotted constructs

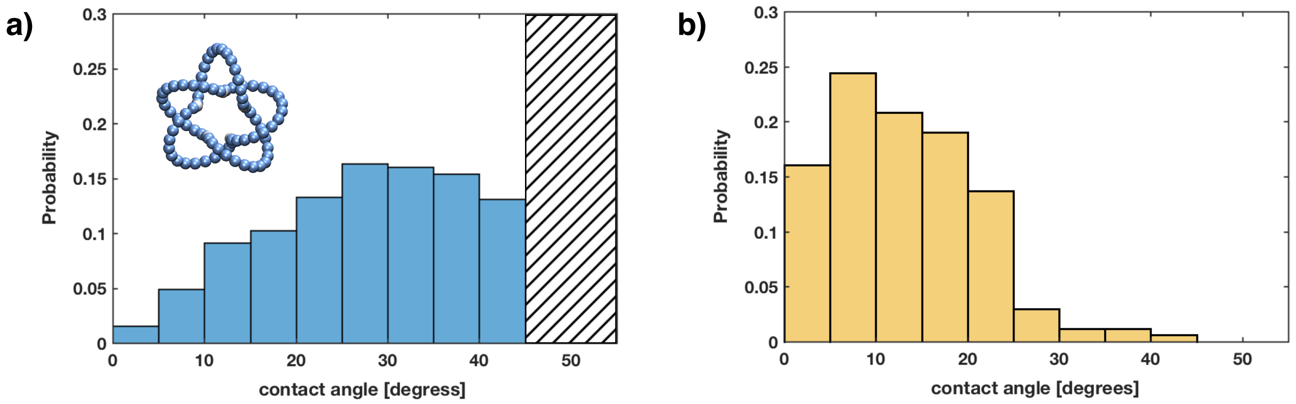

Supplementary Figure 4: **(a)** Probability distribution of the contact angle between consecutive templates in the Monte Carlo-generated cyclic-symmetric 5_1 knots made of 5 templates (data cumulated over all explored template shapes). Note that angles larger than $\pi/4$, or 45 degrees are disallowed and hence are not populated. The analogous distribution, but pertaining to molecular dynamics simulations, are shown in panel **(b)**. In this case, the shape of the distribution is controlled by the steric and patchy interactions of the templates which promote the collinearity of the contacting templates' ends. Because of these smaller contacting angles, which are much lower than needed to establish 5_1 knots - see panel (a)- , the formation of 5_1 knots in MD assembly simulations is suppressed.

Supplementary Note 5. Monte Carlo cyclic-symmetry score

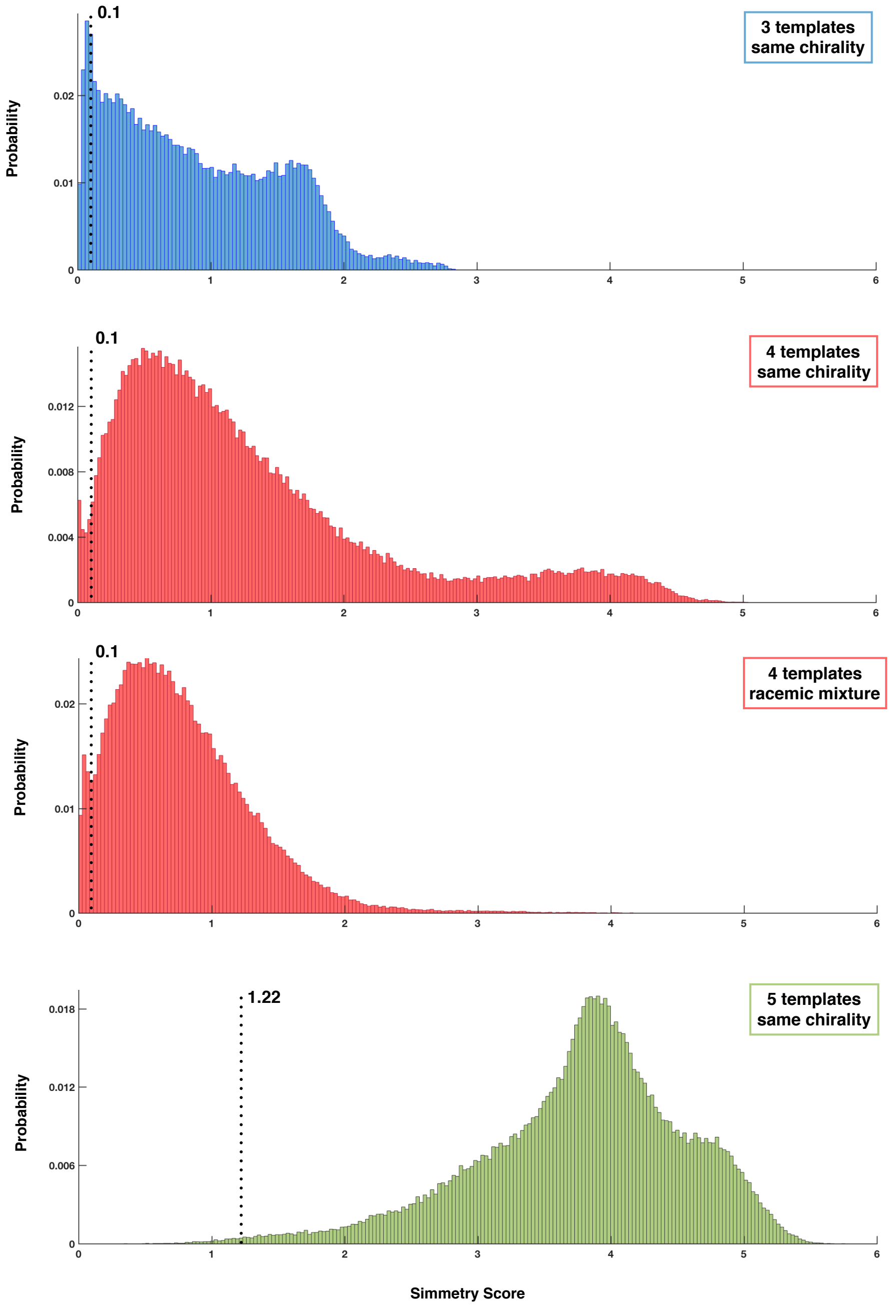

Supplementary Figure 5: Probability distribution of the cyclic-symmetry score for Monte-Carlo sampled constructs made of $n_T = 3$, 4, and 5 templates of same or different chiralities, as indicated. Each distribution is cumulated over all considered templates' shapes. The symmetry score is computed as the root mean square deviation (RMSD) of the structural alignment of a construct with its circular permutant with the best (Kabsch) structural alignment. The best alignment is searched over all cyclic permutations of the beads indices with an indexing shift at least equal to half the templates' length (number of beads). For three and four templates, the presence of cyclic-symmetric constructs is signalled by a peak or shoulder at low values of the score. The cutoff value for the score used to select such instances is marked with a dashed line. For five templates, no peak is discernible and therefore we took the RMSD cutoff value (again indicated with a dashed line) as the largest RMSD below which all constructs are cyclic-symmetric. By this we mean that their geometry can be regularised into a cyclic-symmetric shape with only minor adjustments.

Supplementary Note 6. Effective bonding potential

The nominal strength of the bonding potential is controlled by the adimensional parameter C_p. In our simulations we set $C_p = 25$, so that the depth of the Gaussian well between two patches is equal to 25 $K_B T$. The effective unbonding barrier is appreciably smaller than this, as it is clarified by computing the effective free-energy profile, $F(r)$, of two patchy particles as a function of their distance r,

$$F(r) = -K_B T \log(Z(r)) \tag{1}$$

where $Z(r)$ is the canonical partition function integrated over the degrees of freedom, $\{\theta, \phi, \theta', \phi'\}$ defining the relative orientation of the patchy particles at the given distance r. Apart from a prefactor, contributing only to an additive shift of $F(r)$, $Z(r)$ is given by:

$$Z(r) = \left(\frac{r}{\sigma}\right)^2 \int_{\cos\theta=-1}^{\cos\theta=+1} \int_{\phi=0}^{\phi=2\pi} \int_{\cos\theta'=-1}^{\cos\theta'=+1} \int_{\phi'=0}^{\phi'=2\pi} d\cos(\theta)\ d\cos(\theta')\ d\phi\ d\phi'\ e^{-\left[U^{patchy}(r)+U^{LJ}(r,\theta,\phi,\theta',\phi')\right]/K_B T} \tag{2}$$

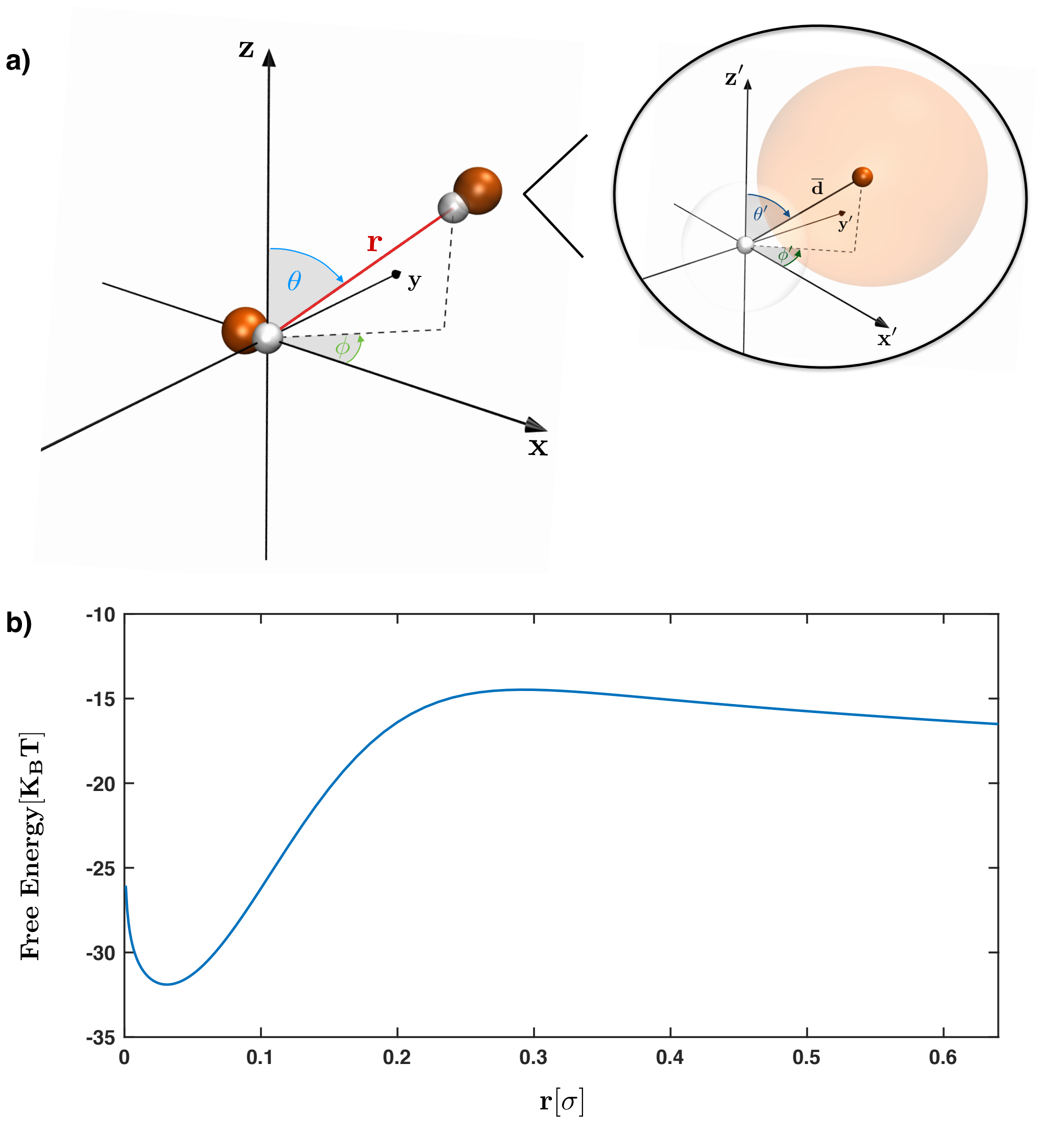

Supplementary Figure 6: **(a)** Representation of the degrees of freedom $(\theta, \phi, \theta', \phi')$ defining the relative orientation of two patchy particles at a distance r. **(b)** Free-energy profile, $F(r)$, obtained by numerical integration for $C_p = 25$.

where θ and ϕ are the radial and azimuthal angles of the second patch defined in the Cartesian frame centered in the first patch and with x axis oriented along the principal axis of the first patchy particle itself, see Fig. 6a. The other parameters, θ' and ϕ' are instead the radial and azimuthal angles that specify the orientation of the center of the second patchy particle in respect to its patch, see inset of Fig. 6a.
The bead-patch distance is fixed and equal to $\overline{d} = 2^{1/6}\sigma/2$.

Numerical integration of $Z(r)$ yields (up to an additive constant) the free energy profile shown in Fig. 6b.

One sees that due to the interplay of entropic and enthalpic effects, the effective barrier for breaking a bond is smaller than 25 K_BT, and specifically it is equal to about 18 K_BT.

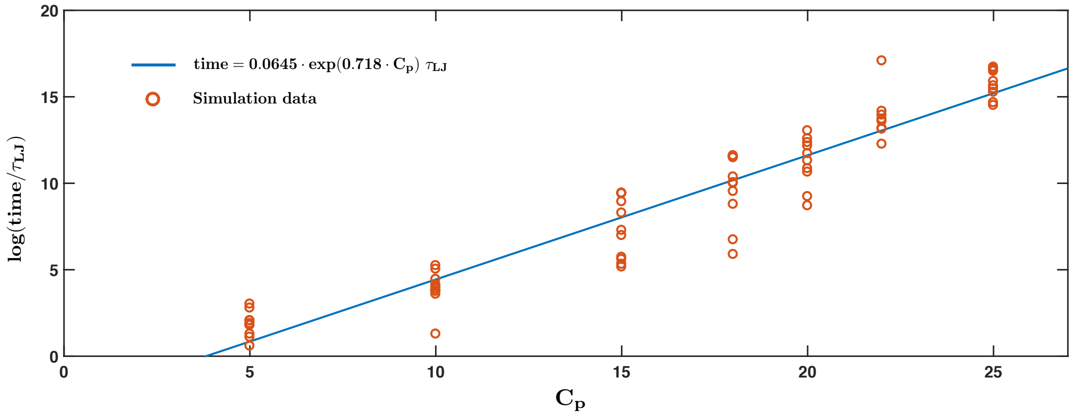

Supplementary Figure 7: Natural logarithm of the time required to break the bond between two patchy helical templates as a function of the strength of the patch-patch potential C_p. Results from simulations are represented with orange circles, while linear fit is represented with a continuous blue line: $time = 0.0645 \cdot \exp(0.718 \cdot C_p)\tau_{LJ}$.

The result is consistent with the actual bond-breaking kinetics for two patchy helical templates, as it is shown in Fig. 7. The semi-log plot shows the C_p dependence of the time required to break the bond between two initially-contacting templates during various MD simulations (ten per each C_p value). The best fit line in Fig. 7 is $time \propto \exp(0.718 \cdot C_p)\,\tau_{LJ}$. For $C_p = 25$, this yields the effective barrier $0.178 \cdot 25\ K_BT \sim 18\ K_BT$. The associated detached time is of the order of $4,000,000\ \tau_{LJ}$, which is about 20 times larger than the typical duration of our production runs.

Supplementary Note 7. Enumeration of braid patterns

The tables below list various single-component knots (i.e. not links) obtainable for various (n_T, n_S) combinations, including few beyond those covered by Fig. 4 of the main text, e.g. $(9,2)$, $(8,3)$ and $(10,3)$. The $(7,4)$ pair is not included, due to the excessively large number of braid combinations yielding knotted patterns that exceed the complexity of tabulated knot types (available for prime components of up to 16 crossings). Knots of up to 10 crossings are denoted with the conventional Rolfsen notation. More complex knots are labelled with the Thistlethwaite notation, except for specific instances of torus knots, for which we use the conventional $T(n_1, n_2)$ notation, and very complex topologies which we fingerprint with their Dowker code.

For each (n_T, n_S) combination, the $2^{n_T(n_S-1)}$ possible braid patterns are subdivided according to several criteria. First we consider the number of crossings projected in the plane orthogonal to the axis of cyclic symmetry, which is clearly an upper bound to the minimal crossing number. Next, we separate braids that admit a closed cyclic-symmetric arrangment from those that do not. The latter are not the main focus of the study and hence, for simplicity, are not included for the more complex cases of $(8,3)$ and $(10,3)$. The symmetric braids are then grouped by the order of their cyclic symmetry. The number of linear braid patterns associated to a given knot type is shown in the second column.

$(\mathbf{n_T = 3, n_S = 2})$

3 projected crossings

Topology	# possible braid rep.
**C_3 - symmetric**	
3_1	2
**Non-symmetric**	
0_1	6

$(\mathbf{n_T = 4, n_S = 3})$

8 projected crossings

Topology	# possible braid rep.
**C_4 - symmetric**	
8_{18}	2
8_{19}	2
**C_2 - symmetric**	
3_1	8
4_1	4
**Non-symmetric**	
0_1	88
3_1	64
5_1	16
5_2	32
6_3	16
8_{20}	16
3_1#3_1	8

$(\mathbf{n_T = 5, n_S = 2})$

5 projected crossings

Topology	# possible braid rep.
**C_5 - symmetric**	
5_1	2
**Non-symmetric**	
0_1	20
3_1	10

$(\mathbf{n_T = 5, n_S = 3})$

10 projected crossings

Topology	# possible braid rep.
**C_5 - symmetric**	
10_{123}	2
10_{124}	2
**Non-symmetric**	
0_1	330
3_1	200
4_1	60
5_1	60
5_2	60
6_2	80
6_3	20
8_9	20
8_{17}	20
8_{19}	20
8_{20}	20
8_{21}	60
10_{141}	20
10_{155}	10
3_1#3_1	40

Supplementary Figure 8: Symmetric and non-symmetric knots for the following (n_T, n_S) pairs: $(3, 2)$, $(4, 3)$, $(5, 2)$, $(5, 3)$. The number of linear braid patterns associated to a given knot type is shown in the second column.

$(\mathbf{n_T = 5, n_S = 4})$

15 projected crossings

Topology	# possible braid rep.
**C_5 - symmetric**	
$15a_{84903}$	2
$15n_{41185}$	2
$15n_{166130}$	4
**Non-symmetric**	
0_1	2910
3_1	3130
4_1	370
5_1	1480
5_2	2360
6_1	100
6_2	140
6_3	1200
7_1	200
7_2	440
7_3	520
7_4	420
7_5	500
7_6	280
7_7	20
8_7	380
8_8	660
8_{10}	680
8_{13}	320
8_{15}	180
8_{16}	140
8_{18}	80
8_{19}	310
8_{20}	1460
8_{21}	50
9_{28}	60
9_{31}	140
9_{33}	40
9_{42}	20
9_{43}	40
9_{44}	120
9_{45}	160
9_{46}	10
9_{48}	90
9_{49}	100
10_{112}	80
10_{114}	40
10_{122}	60
10_{124}	60
10_{125}	280
10_{126}	240
10_{128}	120
10_{129}	160
10_{130}	280
10_{132}	360
10_{134}	120
10_{135}	520
10_{139}	50
10_{140}	140
10_{142}	60
10_{143}	180
10_{145}	80
10_{146}	10
10_{148}	320
10_{151}	440
10_{153}	520
10_{156}	40
10_{157}	20
10_{159}	40
10_{160}	40
$10_{161}=10_{162}$	140
10_{164}	40
10_{165}	80
$11a_{171}$	40
$11n_{12}$	40
$11n_{23}$	80
$11n_{24}$	160
$11n_{39}$	60
$11n_{41}$	40
$11n_{46}$	80
$11n_{50}$	20
$11n_{54}$	160
$11n_{56}$	40
$11n_{61}$	120
$11n_{65}$	80
$11n_{71}$	40
$11n_{78}$	20
$11n_{82}$	40
$11n_{94}$	120
$11n_{95}$	40
$11n_{96}$	200
$11n_{98}$	40
$11n_{106}$	180
$11n_{107}$	20
$11n_{118}$	20
$11n_{132}$	40
$11n_{133}$	20
$11n_{145}$	160
$11n_{146}$	40
$11n_{147}$	40
$11n_{148}$	60
$11n_{173}$	20
$11n_{178}$	20
$11n_{179}$	20
$11n_{183}$	40
$11n_{184}$	20
$12n_{121}$	60
$12n_{242}$	20
$12n_{253}$	40
$12n_{309}$	140
$12n_{318}$	160
$12n_{323}$	200
$12n_{328}$	40
$12n_{371}$	140
$12n_{385}$	80
$12n_{425}$	60
$12n_{426}$	20
$12n_{439}$	100
$12n_{443}$	80
$12n_{451}$	80
$12n_{488}$	100
$12n_{548}$	40
$12n_{591}$	40
$12n_{646}$	40

$12n_{702}$	80
$12n_{725}$	10
$12n_{730}$	40
$12n_{749}$	60
$12n_{811}$	20
$12n_{829}$	20
$12n_{835}$	40
$12n_{868}$	10
$13n_{225}$	40
$13n_{288}$	80
$13n_{501}$	60
$13n_{519}$	20
$13n_{584}$	40
$13n_{586}$	20
$13n_{592}$	120
$13n_{601}$	40
$13n_{603}$	40
$13n_{606}$	120
$13n_{608}$	80
$13n_{1192}$	20
$13n_{1644}$	40
$13n_{1692}$	80
$13n_{1716}$	40
$13n_{1718}$	40
$13n_{1719}$	80
$13n_{1724}$	80
$13n_{1727}$	40
$13n_{1734}$	20
$13n_{1735}$	40
$13n_{1739}$	40
$13n_{1931}$	120
$13n_{1945}$	40
$13n_{1957}$	20
$13n_{2303}$	40
$13n_{2436}$	40
$13n_{2442}$	40
$13n_{2491}$	40
$13n_{2787}$	120
$13n_{3023}$	40
$13n_{3351}$	20

$13n_{3393}$	80
$13n_{3582}$	40
$13n_{3611}$	40
$13n_{3956}$	40
$13n_{3958}$	40
$13n_{3969}$	40
$13n_{3973}$	40
$13n_{3978}$	20
$13n_{3979}$	40
$13n_{3982}$	40
$13n_{3998}$	40
$13n_{4003}$	40
$13n_{4035}$	60
$13n_{4051}$	10
$13n_{4079}$	60
$13n_{4080}$	60
$13n_{4634}$	20
$13n_{5018}$	10
$14n_{6174}$	10
$14n_{21472}$	20
$14n_{22172}$	20
$14n_{22583}$	20
$14n_{23344}$	20
$15n_{40180}$	20
$15n_{40184}$	40
$15n_{40185}$	20
$15n_{41127}$	20
$15n_{41189}$	20
$15n_{41193}$	60
$15n_{41223}$	20
$15n_{41235}$	40
$15n_{43517}$	30
$15n_{45460}$	10
$15n_{46935}$	40
$15n_{46936}$	20
$15n_{47800}$	40
$15n_{48957}$	20
$15n_{49035}$	40
$15n_{51709}$	20
$15n_{52941}$	10

$15n_{52944}$	20
$15n_{53947}$	20
$15n_{53948}$	20
$15n_{56026}$	20
$15n_{59004}$	40
$15n_{59005}$	20
$15n_{59007}$	20
$15n_{71113}$	20
$15n_{107628}$	40
$15n_{124826}$	40
$15n_{125031}$	40
$15n_{125991}$	20
$15n_{126002}$	20
$15n_{126008}$	20
$15n_{126010}$	40
$15n_{126011}$	20
$15n_{126024}$	10
$15n_{127000}$	20
$15n_{127094}$	10
$15n_{127330}$	10
$15n_{127609}$	20
$15n_{127610}$	20
$15n_{127630}$	40
$15n_{127654}$	40
$15n_{163844}$	20
$15n_{163860}$	20
$15n_{166131}$	10
$15n_{166806}$	10
$3_1\#3_1$	730
$3_1\#4_1$	60
$3_1\#5_1$	280
$3_1\#5_2$	400
$3_1\#6_3$	80
$3_1\#8_{20}$	60
$3_1\#8_{19}$	40
$3_1\#3_1\#3_1$	10

Supplementary Figure 9: Symmetric and non-symmetric knots for the following (n_T, n_S) pairs: $(5, 4)$. The number of linear braid patterns associated to a given knot type is shown in the second column.

$(\mathbf{n_T = 7, n_S = 2})$

7 projected crossings

Topology	# possible braid rep.
**C_7 - symmetric**	
7_1	2
**Non-symmetric**	
0_1	70
3_1	42
5_1	14

$(\mathbf{n_T = 7, n_S = 3})$

14 projected crossings

Topology	# possible braid rep.
**C_7 - symmetric**	
$14a_{19470}$	2
$14n_{21881}$	2
**Non-symmetric**	
0_1	2688
3_1	2884
4_1	224
5_1	1260
5_2	1400
6_2	168
6_3	896
7_1	280
7_3	280
7_5	560
8_7	336
8_9	28
8_{10}	336
8_{16}	168
8_{17}	28
8_{18}	112
8_{19}	280
8_{20}	868
8_{21}	140
10_{17}	28
10_{48}	56
10_{79}	28
10_{91}	56
10_{99}	14
10_{104}	28
10_{109}	28
10_{112}	168
10_{118}	28
10_{124}	70
10_{125}	196
10_{126}	140
10_{139}	112
10_{141}	28
10_{143}	280
10_{148}	280
10_{155}	14
10_{157}	70
10_{159}	140
$10_{161}=10_{162}$	168
$12a_{819}$	28
$12a_{1209}$	28
$12a_{1211}$	28
$12n_{242}$	28
$12n_{468}$	56
$12n_{675}$	56
$12n_{708}$	56
$12n_{709}$	56
$12n_{721}$	28
$12n_{749}$	112
$12n_{751}$	28
$12n_{829}$	140
$14n_{21882}$	28
$14n_{24169}$	28
$14n_{27039}$	28
$14n_{27120}$	14
$3_1\#3_1$	588
$3_1\#5_1$	168
$5_1\#5_1$	14

Supplementary Figure 10: Symmetric and non-symmetric knots for the following (n_T, n_S) pairs: $(7, 2)$, $(7, 3)$. TThe number of linear braid patterns associated to a given knot type is shown in the second column.

$(\mathbf{n_T = 8, n_S = 3})$

16 projected crossings

Topology	# possible braid rep.
**C_8 - symmetric**	
$16a_{379778}$	2
$16n_{783154}$	2
**C_4 - symmetric**	
8_{18}	4
8_{19}	8
**C_2 - symmetric**	
3_1	48
4_1	40
8_5	48
8_{19}	16
$12n_{725}$	72
$12n_{750}$	32
$12a_{1229}$	16
$12a_{1288}$	8
$16n_{998580}$	16
**Non-symmetric**	
...	

$(\mathbf{n_T = 9, n_S = 2})$

9 projected crossings

Topology	# possible braid rep.
**C_9 - symmetric**	
9_1	2
**C_3 - symmetric**	
3_1	6
**Non-symmetric**	
0_1	252
3_1	162
5_1	72
7_1	18

$(\mathbf{n_T = 10, n_S = 3})$

20 projected crossings

Topology	# possible braid rep.
**C_{10} - symmetric**	
T(10,3)	2
putative 20-crossing knot #1	2
**C_5 - symmetric**	
10_{123}	4
10_{124}	8
**C_2 - symmetric**	
3_1	210
8_5	80
8_{18}	60
8_{19}	120
$12a_{1210}$	80
$12a_{1229}$	20
$12a_{1288}$	10
$12n_{725}$	60
$12n_{750}$	60
$12n_{888}$	30
$16a_{377123}$	20
$16a_{377444}$	20
$16n_{783154}$	20
$16n_{998580}$	20
$16n_{1003403}$	60
putative 20-crossing knot #2	20
putative 20-crossing knot #3	10
**Non-symmetric**	
...	

	Dowker Code of putative 20-crossing knots for $(\mathbf{n_T = 10, n_S = 3})$
putative 20-crossing knot #1	14 16 18 20 22 24 26 28 30 32 34 36 38 40 2 4 6 8 10 12
putative 20-crossing knot #2	10 -12 14 -18 -38 26 -28 30 -6 -32 -34 -36 40 -2 4 16 -20 -22 -8 24
putative 20-crossing knot #3	10 14 -16 20 24 28 30 32 -34 6 -36 8 38 40 12 2 -4 -18 -22 26

Supplementary Figure 11: Symmetric and non-symmetric knots for the the following (n_T, n_S) pairs: $(9, 2)$, $(8, 3)$ and $(10, 3)$. The number of linear braid patterns associated to a given knot type is shown in the second column. Three knot types with 20 projected crossings are fingerprinted by their Dowker code, which could not be further simplified algebraically with the Knotscape software package.
