# Supplementary material for: Discovering privileged topologies of molecular knots with self-assembling models
Source: Nat Commun. 2018 Aug 3;9:3051. doi: 10.1038/s41467-018-05413-z (PMC6076300; doi:10.1038/s41467-018-05413-z)
Supplement: Supplementary file 2 — Description of Additional Supplementary Files [file 41467_2018_5413_MOESM2_ESM.pdf]

Description of Additional Supplementary Files

File Name: Supplementary Dataset 1
Description: Cartesian coordinates for the representative knotted constructs shown in Figures 2 and 3 of the main text.
